# Volcanic eruptions are triggered in static dilatational strain fields generated by large earthquakes

**DOI:** 10.1038/s41598-021-96756-z

**Published:** 2021-08-26

**Authors:** Takeshi Nishimura

**Affiliations:** Department of Geophysics, Graduate School of Science, Tohoku University, Sendai, 980-8578 Japan

**Keywords:** Natural hazards, Solid Earth sciences, Volcanology

## Abstract

Although data catalog analyses have confirmed that volcanic eruptions are triggered by large earthquakes, the triggering mechanism has been under discussion for many decades. In the present study, recent earthquake and volcanic data from the past 35–55 years were analyzed, and it was demonstrated for the first time that the likelihood of new eruptions increases two to three times in the 5–10 years following large earthquakes for volcanoes where the generated static dilatational strain exceeds 0.5 µ, which may, for example, activate gas bubble growth and thereby generate a buoyant force in the magma. In contrast, the eruption likelihood does not increase for volcanoes that are subjected to strong ground motion alone, which affect the magma system and volcanic edifice. These results indicate that we can evaluate the likelihood of triggered eruptions and prepare for new eruptions when a large earthquake occurs.

## Introduction

Large earthquakes are considered to sometimes trigger eruptions at nearby volcanoes because there have been numerous cases of a close spatiotemporal occurrence of the two types of event. Previous studies using global catalogs including data from the sixteenth century to the present have demonstrated such coincidences to be within a few days and several hundred kilometers^1,2^. Analyses of recent global catalogs alone indicate that triggered eruptions continue for several years after the occurrence of large earthquakes^3,4^. Many analyses on the regional and local scales have also shown spatial and temporal correlations between earthquakes and eruptions^5^. These previous results based on data catalog analyses provide evidence that large earthquakes can sometimes trigger eruptions of nearby volcanoes.

To understand such empirical interactions between earthquakes and volcanic eruptions, triggering mechanisms have been discussed for many decades^6–10^. The proposed mechanisms are static dilatational strain, static compressional strain, and dynamic strain. Static dilatational strain may activate gas bubble growth in magma, introducing a buoyant force that drives the magma upward. Dike opening may also be induced by extensional stress. An example of this phenomenon is the 1707 Hoei eruption at Mt. Fuji, Japan, which occurred 49 days after the Hoei earthquake with a magnitude of 8^11^. Static compressional strain may squeeze the magma in a chamber to the ground surface. The 1991 Pinatubo eruption may be an example of this mechanism; with a volcanic explosivity index (VEI) of 6, this eruption was the largest in the twentieth century, and it occurred one year after the 1990 M7.8 Luzon earthquake in the Philippines^12^. Finally, dynamic strain generated by seismic waves, such as strong ground motion and surface waves propagating over a long distance that can even exceed 1000 km, can activate volcanoes. Dynamic strain is generally not directly instrumentally recorded but is converted from the ground velocity recorded by seismometers by assuming the S-wave velocity in the medium^13^. Because strong ground motion and surface waves generate dynamic stress in magma chambers and volcano edifices with a period ranging from 10 ms to a few hundred seconds, various types of triggering mechanisms have been proposed. As summarized in Seropian et al.^14^, they include (1) volatile processes involving bubble nucleation and growth, advective overpressure associated with bubble rise, and/or falling crystalline roofs facilitating vesiculation^9,15–17^; (2) resonance processes such as sloshing and edifice resonance that may activate volatile migration due to foam collapse, increased degassing and vesiculation in magma, or melt and volatile migration inside the volcanic edifice^18^; and (3) destabilization in the hydrothermal and geothermal systems that may introduce depressurization of the magma system and trigger eruptions^9,19^.

These triggering mechanisms have been discussed on the basis of theoretical considerations and experimental laboratory results, and the interaction between large earthquakes and volcanoes have been quantitatively evaluated at regional and local scales. However, it is not easy to objectively clarify which mechanism plays a main role because volcanoes located near the hypocenters of large earthquakes are generally subject to both large static strain and strong ground motion; as a result, the mechanism is still under discussion. The present study carefully examines the recent global data catalog of large earthquakes and volcanic eruptions to determine which triggering mechanism is most likely: static dilatational strain, static compressional strain, or strong ground motion. By systematically analyzing global data instead of targeting individual volcanoes or regions, the present study quantitatively evaluates the occurrence possibility and frequency of eruptions that can be triggered by large earthquakes. The results enable us to prepare for a new eruption caused by a large earthquake based on the average triggering possibility.

## Results

### Static and dynamic strain caused by large earthquakes near volcanoes

Continuous instrumental observations of earthquakes and systematic collection of volcanic eruption data over recent decades has provided a rich bank of reliable data spanning several tens of years. The seismic data used in the present study are the occurrence time, centroid location, seismic moment, and mechanism of large earthquakes from the global centroid moment tensor (CMT) catalog provided by Columbia University, which dates back to 1976^20^. Volcanic eruption data are taken from the database of the Global Volcanism Program operated by the Smithsonian Institute^21^. The eruption start and end dates as well as the VEI, which represents the magnitude of the eruption^22^, are listed in this database. Although volcanic eruptions sometimes continue over several days, months, or years, only the start time is examined in the present study because the triggering of a new eruption is the main focus here. To avoid contamination from missing data, only earthquakes with magnitudes *M* of at least 6 and volcanic eruptions with VEIs of at least 2 are analyzed (see “Methods” section). The yearly numbers of eruptions and earthquakes with VEIs and magnitudes meeting these criteria are approximately 20 and 100, respectively.

**Figure 1:**
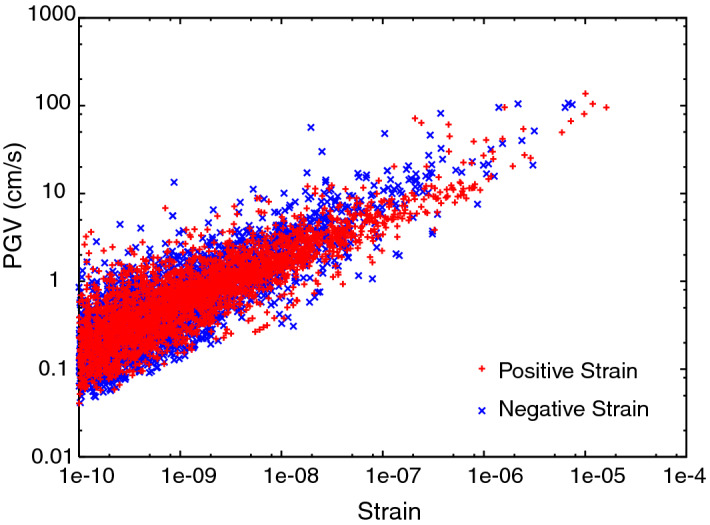
PGV plotted against static strain at volcanoes within distance of 500 km from large earthquakes.

Figure 1 shows the peak ground velocity (PGV) for strong ground motion at volcanoes that erupted within 10 years before or after large earthquakes plotted against the static strain (ε). The static dilatational and compressional strains are theoretically calculated using the centroid moment tensor solutions from the CMT catalog (see “Methods” section). The PGV is calculated from the seismic magnitude and distance from the seismic fault (see “Methods” section). Data for volcanoes with a horizontal distance of at most 500 km from a large earthquake are included in the plot. The static strain ranges from approximately − 10 to 10 µ, where positive and negative values represent dilatation and compression, respectively. The maximum predicted PGV is approximately 100 cm/s, which corresponds to a dynamic strain of approximately 500 µ (see “Methods” section). Both are mainly dependent on the magnitude of the earthquake and the distance from the seismic fault. The PGV increases with increasing static strain magnitude, although the decay rate for the PGV with increasing distance is smaller than that for the static strain (Supplementary Figure 1) because the amplitude of the static strain rapidly decreases in proportion to the inverse of the cube of the distance whereas that of the PGV is inversely proportional to the square of the distance.

### Number of eruptions before and after large earthquakes

The present study compares the numbers of eruptions before and after large earthquakes, following the methods presented in many previous studies^1–3^ (see “Methods” section). The occurrence time for each large earthquake is set to zero, and the occurrence times for volcanic eruptions are aligned with a lag time from the occurrence time for large earthquakes. Negative and positive lag times represent before and after the occurrence of the corresponding large earthquake, respectively. To evaluate long-term effects, lag times of ± 10 years from the occurrence of large earthquakes are examined. As a result, the earthquake data for 35 years from 1976 to 2010 and the eruption data catalog for 55 years from 1966 to 2020 are used.

**Figure 2:**
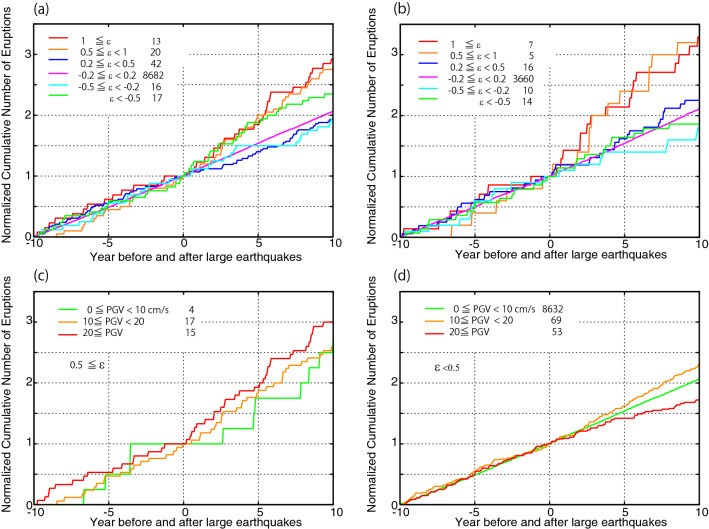
Cumulative number of volcanic eruptions before and after large earthquakes for various static strains and PGVs normalized by the number at 0 years. (**a**) Distributions for various strains under no PGV constraints for all volcano data. (**b**) Same as (**a**) but for only those volcanoes that erupted fewer than 10 times in the past 55 years. (**c**) Distributions for various PGVs for static dilatational strain of at least $0.5$ µ. (**d**) Distributions for various PGVs under static strain of less than $0.5 \mu$. The numbers in the legends are the cumulative numbers of eruptions at 0 years for each strain and PGV condition.

Figure 2a shows the cumulative number of eruptions before and after large earthquakes for six static strain ranges. No constraint is given on the PGV. The total number of eruptions are different for the six static strain ranges, hence each cumulative number is normalized by the number at a lag time of zero to enable better comparison of the results obtained under different criteria. The cumulative number before large earthquakes (lag times from − 10 to 0 years) does not show significant differences across the static strain ranges and increases monotonically with lag time. After large earthquakes (lag times from 0 to 10 years), the cumulative number for a dilatational strain of 0.5 µ and above deviates from that for the other strain ranges. The occurrence rate for eruptions after large earthquakes was approximately double that before, for a lag time from 0 to 5–10 years. In contrast, the cumulative number for dilatational strain below 0.5 µ does not significantly increase after large earthquakes, as the occurrence rate before and after large earthquakes is almost the same. Compressional strains with magnitudes above 0.5µ (i.e., strains below − 0.5 µ) show an increase in the eruption occurrence rate of approximately 50% for roughly 5 years after the occurrence of large earthquakes. However, as shown below, this does not occur consistently.

Some volcanoes show frequent eruptions with VEIs of 2 or more for the 55 years from 1966 to 2020. The Bezymianny, Klyuchevskoy, Etna, and Ulawn volcanoes erupted at least 20 times, and 34 volcanoes erupted at least 10 times (Supplementary Table 1). To examine whether these frequently erupting volcanoes may bias the results, only eruptions at volcanoes with fewer than 10 volcanic eruptions over the considered 55-year span are analyzed (Fig. 2b). The results are similar to the case using all volcanoes, with an occurrence rate after large earthquakes of two to three times that before. However, the occurrence rate for large compressive strain (below − 0.5 µ) does not show any increase after large earthquakes, suggesting that the frequent eruptions affected the occurrence rate analysis for compressive strain.

To examine whether these increases in the eruption occurrence rate are significant, a simple test is conducted using simulated data in which the occurrence times of large earthquakes are randomly set in the catalogs while the centroid locations and source mechanisms of earthquakes and the eruption dates are kept the same as in the original data. The results obtained from 1000 simulations show that only approximately 1% of the simulated data can reproduce the increase of the occurrence rate for approximately 5–10 years after large earthquakes when the strain exceeds $0.5$µ (Supplementary Table 2, Supplementary Figure 2). The simulations for the other static strains, including strains below − 0.5 µ, show that the empirical rates are distributed in the middle range of the simulation results. These simulations strongly support the observed increase in eruptions at volcanoes subject to dilatational static strain with an amplitude exceeding $0.5$µ after large earthquakes. Furthermore, no significant evidence points to any contribution of compressional strain to increasing the likelihood of eruption. Thus, volcanic eruptions occurring in compressional strain fields can be explained by random occurrence.

Figure 2c and d show the cumulative number of eruptions for different PGVs for ε ≥ 0.5 µ and ε < 0.5 µ. For ε  ≥ 0.5 µ, some increase in the eruption occurrence rate after large earthquakes is observed for all PGV ranges, although the number of eruptions with PGV < 10 cm/s is small. For ε < 0.5 µ, large PGVs of ≥ 20 cm/s tend to decrease by about 20–30%, whereas the occurrence rate increases by approximately 20% after large earthquakes with intermediate PGVs of 10–20 cm/s. The simulation results indicate that these changes in the eruption occurrence rate are within the range that can be explained by random occurrences. This suggests that strong ground motion (dynamic strain) does not significantly contribute to the triggering of eruptions.

To further confirm that the main mechanism triggering volcanic eruptions is dilatational strain and not strong ground motion, the numbers of eruptions and erupting volcanoes are evaluated for cases with large dilatational strain and small PGV, which are rare (Table 1). Globally, 3591 earthquakes with a magnitude of at least 6 occurred in the 35 years from 1976 to 2010, and only 10 of these earthquakes satisfied the conditions of *ε*  ≥ 0.5 µ and PGV < 10 cm/s at 9 volcanoes. For the 5 and 10 years preceding these earthquakes, 2 and 4 volcanoes erupted, respectively, whereas 3 and 4 erupted in the 5 and 10 years after, respectively; the number of eruptions also increase from 2 to 3 and from 4 to 6, respectively. Although there are only 9–10 volcanoes that satisfy the conditions of *ε*  ≥ 0.5 µ and PGV < 10 cm/s, the numbers of eruptions and erupting volcanoes before large earthquakes are equal to or larger than those after large earthquakes. In Table 1, the other two cases are shown. As suggested by Fig. 1, the cases with *ε*  ≥ 0.5 µ and PGV ≥ 10 cm/s demonstrate that the numbers of both erupted volcanoes and eruptions increase after large earthquakes, while the cases with *ε* < 0.5 µ and PGV ≥ 10 cm/s do not show significant changes.

**Table 1:** Number of eruptions and volcanoes that erupted.

Years before & after a large earthquake*	Volcanoes satisfying the conditions	Volcanoes that erupted**		Eruptions	
		Before	After	Before	After
${\varvec{\varepsilon}}\ge 0.5{\varvec{\mu}}$**, PGV < 10 cm/s**					
5	10	2	3 (1)	2	3
10	9	4	4 (3)	4	6
${\varvec{\varepsilon}}\ge 0.5$**µ, PGV ≥ 10 cm/s**					
5	136	11	16 (7)	15	30
10	101	13	21 (10)	28	55
${\varvec{\varepsilon}}<0.5$ **µ, PGV ≥ 10 cm/s**					
5	287	42	43(18)	72	74
10	244	58	60(32)	124	130

*10 years: CMT 1976–2010. 5 years: CMT 1976–2015.

**Numbers in parentheses represent the number of volcanoes that erupted both before and after large earthquakes.

The results shown in Fig. 2 and Table 1 strongly support the conclusion that new eruptions are triggered by static dilatational strain exceeding 0.5 µ and not by large strong ground motion in the absence of such strain. In Supplementary Table 3, the earthquakes inducing static dilatational strain above 0.5 µ at nearby volcanoes are listed with triggered eruptions. Note that about a half of the eruptions is considered to have randomly occurred without triggering by large earthquakes, because the cumulative number of eruptions constantly increases with time before large earthquakes.

## Discussion

### Triggering mechanism of volcanic eruptions by large earthquakes

Many of the volcanoes did not erupt even when external triggering forces are given by large earthquakes. This is considered that such volcanoes were not close to being ready to erupt^23^. But it is still difficult to quantitatively evaluate the readiness to erupt at active volcanoes from analyses of seismic, geodetic and/or other instrumentally recorded data. Hence, the present study instead examines eruptive activity at volcanoes with triggered eruptions, which is defined as an eruption that occurred at a volcano where a static dilatational strain of at least 0.5 µ was introduced by a large earthquake.

**Figure 3:**
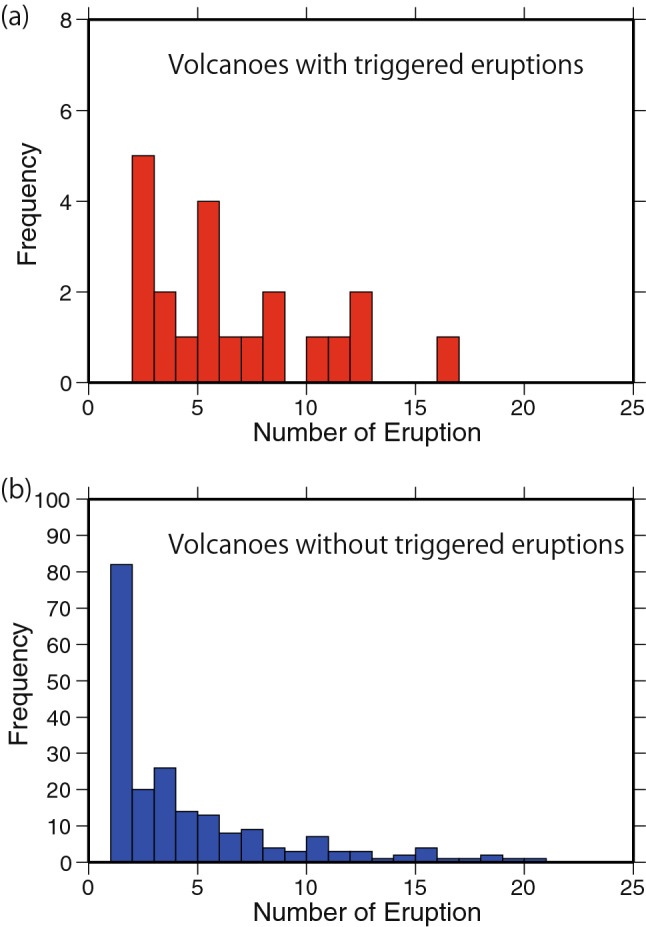
Frequency of volcanoes with a given number of eruptions over the 55 studied years for volcanoes (**a**) with and (**b**) without eruptions triggered by large earthquakes. This figure was created by Generic Mapping Tools (GMT) (version 4.5.5, https://www.soest.hawaii.edu/gmt/)^35^.

Figure 3 shows the frequency of volcanoes with a given number of eruptions over the 55 years from 1966 to 2020. The volcanoes with triggered eruptions show distributions skewed towards larger numbers of eruptions in comparison with those without triggered eruptions, and many volcanoes without triggered eruptions erupted only once during the 55-year span. These distributions demonstrate that the volcanoes that have frequently erupted are more easily triggered than the volcanoes that have more rarely erupted.

**Figure 4:**
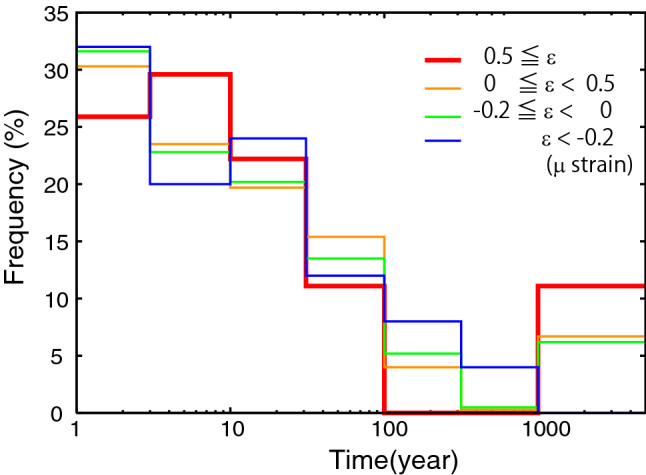
Normalized distributions of lag time from most recent eruption to occurrence of large earthquakes at volcanoes under different static strains.

Next, the present study examines how long volcanoes have been dormant before being triggered. Figure 4 shows the distributions of the lag times between the occurrence of large earthquakes and the most recent eruptions with VEI of at least 2 at triggered volcano. The frequencies are obtained for several static strain ranges. For strains of at least 0.5 µ, approximately 50% and 90% of the volcanoes had erupted in the 10 and 100 years preceding the large earthquake, respectively. The others, approximately 10% of the volcanoes, had not erupted in the 1000 years preceding large earthquakes; the volcanoes are Korovin in Aleutian and Sinabun in Indonesia. The former has several uncertain eruptions in 1951, 1953, 1976, 1986, a confirmed eruption with VEI 0 in 1973, and a confirmed eruption with unknown VEI in 1907, but no eruption with VEI of at least 2^21^. These reports suggest some magma activities before the triggered eruption. On the other hand, the latter has no record of eruption and significant volcanic activity^21^, which implies that such dormant volcanoes are also triggered by static dilatational strains caused by large earthquakes. The distributions of the lag times for the other strain ranges, even for compressional strains, are similar to those for $\varepsilon \ge$ 0.5 µ within approximately 5–10%. This suggests that the triggered and non-triggered volcanoes are not discriminated from the characteristics of previous eruptive activities.

The static dilatational strain plays a role in triggering gas bubble growth to promote upward magma migration due to buoyancy, because gas bubble growth and/or pressurization are promoted in magma when the media surrounding the magma is depressurized^24^. However, if no gas bubbles are present in volcanoes with long quiescent times, the magma may not achieve the necessary buoyancy force to erupt. In such cases, strong ground motions that almost always accompany large static strains may play a role because oscillatory pressure disturbance and fluctuations in magma may nucleate gas bubbles^2^. Seropin et al.^14^ pointed out other triggering mechanisms. The static dilatational strain may also unclamp the pathway of magma to the ground surface from a deeper region^25,26^. Permeability increase in the volcanic edifice caused by the static dilatational strain may allow advection of volcanic fluids. Strong ground motions may also activate these mechanisms.

In the present study, the strain field was evaluated based on the volumetric strain. However, it is known that dikes intrude along the direction parallel to the orientation of maximum stress and there are variations in regional stress directions^6^. Hence, it is necessary to include stress orientations for investigating the triggering mechanism in detail. Furthermore, some previous reports have discussed the relationship between the strain and volcano characteristics such as magma properties and open/closed conduit systems^14,27^. However, because the total number of eruptions is not sufficiently large to discuss the effect of volcano characteristics, continuous efforts to accumulate correct data are necessary to enable us to further deepen our knowledge on the triggering mechanism.

### Occurrence frequency of triggered volcanic eruptions

Although strong ground motion may play a role in triggering eruptions, the static dilatational strain can be used to quantitatively evaluate the increase in the likelihood of a new eruption because strong ground motion often accompanies large static strain. Finally, the present study evaluates how frequently volcanic eruptions are triggered by large earthquakes globally.

Because volcanic eruptions are inferred to be triggered for 5–10 years after a large earthquake, the CMT catalog for 40 years from 1976 to 2015 and volcanic eruption catalogs for 55 years from 1966 to 2020 are analyzed. The results are summarized in Table 2, which gives the number of volcanoes subjected to a dilatational strain of at least 0.5 µ, the number of eruptions that occurred in the 5 and 10 years following large earthquakes, and the number of volcanoes responsible for these eruptions. The strong ground motion is not considered a criterion for these results. Earthquakes with magnitudes of 7 or more induced static dilatational strains of at least 0.5 µ at 135 and 101 volcanoes from 1976 to 2015 (5-year analysis) and 1976 to 2010 (10-year analysis), respectively. Of these volcanoes exceeding the strain threshold, 19 (of 135) and 25 (of 101) erupted in the 5 and 10 years following large earthquakes, respectively. Smaller earthquakes with magnitudes of 6 or more also generated static strain around the centroid, so the number of volcanoes meeting the dilatational stress criterion and that triggered eruptions are slightly higher than for magnitudes of 7 or more. These estimations indicate that, every year, approximately 2–3 volcanoes are likely subjected to dilatational stress caused by large earthquakes exceeding the triggering threshold, and approximately 15–25% of them are expected to erupt in the 5–10 years following a large earthquake. The number of candidates for eruption is not large, but it is worth noting that the present study focused on eruptions with VEIs of at least 2 to avoid missing eruptions. If we consider that the power-law scaling of eruption frequency with VEI is applicable in the range of small VEI, eruptions with a VEI of 1 occur approximately 7 times more often than those with a VEI of 2^28,29^. If such small eruptions are also triggered by static dilatational strain, the likelihood of a smaller eruption is higher than evaluated herein for VEIs of at least 2.

**Table 2:** Number of volcanoes and volcanic eruptions for 5 and 10 years from occurrence of large earthquakes.

Earthquake magnitude	Total number of earthquakes	N_VOL_	Number of eruptions after earthquakes	Number of erupting volcanoes
**5 years**				
$\ge$ 6	4176	146	33	19
$\ge$ 7	350	135	33	19
$\ge 7.5$	120	124	29	17
**10 years**				
$\ge$ 6	3591	110	61	25
$\ge$ 7	350	101	61	25
$\ge 7.5$	120	93	53	23

N_VOL_: Number of volcanoes subject to static dilatational strains of at least $0.5$ µ

Results for 5 years were obtained from analysis of 45 years of CMT data from 1976 to 2015.

Results for 10 years were obtained from analysis of 40 years of CMT data from 1976 to 2010.

## Methods

### Data selection

The reliability of the seismic and volcanic eruption data in the catalog is confirmed by examining (1) whether the frequency distributions of the earthquake magnitudes and VEIs follow power laws, such as that described by the Gutenberg–Richter relation, and (2) their temporally constant occurrence. Earthquakes with a moment magnitude of at least 5 and volcanic eruptions with VEIs of at least $2$ were found to follow power laws (Supplementary Figure 3). The yearly number of earthquakes with magnitudes of 6 or more has been almost constant since 1976, and the yearly number of volcanic eruptions with VEIs of at least $2$ has been nearly constant with slight fluctuations since 1900 (Supplementary Figure 4). To avoid missing data, the present study uses earthquakes with magnitudes of at least 6 and volcanic eruptions with VEIs of at least $2$.

### Counting the number of eruptions

The volcanic eruptions are counted using the following iterative procedure. (1) Earthquakes in the catalog are sorted by time from 1976 to 2010, and all earthquakes with magnitudes below 6 are eliminated. (2) The *i-*th target earthquake ${E}_{i}$ is selected from the data catalog. (3) If an earthquake larger than ${E}_{i}$ occurs within a given time ${T}_{b}$ before ${E}_{i}$, ${E}_{i}$ is judged to be an aftershock and eliminated. (4) The volumetric strain and strong ground motion caused by the earthquake are calculated for all volcanoes within a distance of 500 km from the centroid of the earthquake. (5) Volcanoes satisfying the given volumetric strain and strong ground motion criteria are selected, and the lag time from the occurrence of ${E}_{i}$ to the eruption of the volcano is calculated. (6) Steps (2)–(5) are repeated. (7) The eruption lag times are combined for all large earthquakes selected in Steps (2)–(6). In these calculations, ${T}_{b}$ is set to 14 days, and the lag times are calculated in days. From 1976 to 2010, 3591 earthquakes with a moment magnitude of at least 6 are reported in the CMT catalog. Among them, 3069 earthquakes are selected as target earthquakes.

### Calculation of static strain caused by large earthquakes

The static strain generated at a volcano by a large earthquake is calculated at a depth of 5 km using the algorithm presented by Okada^30^. The latitude, longitude, and depth of the centroid as well as the moment tensor solutions given in the CMT catalog are used for the calculation. A Poisson ratio of 0.25 is assumed. Examples are shown in Supplementary Figure 5.

### Calculation of peak ground velocity generated by large earthquake

Many previous studies have presented methods to calculate the PGV and/or acceleration (PGA) at various regions around the world. Most of them derive empirical equations of the PGV and/or PGA based on strong ground motion data recorded by seismic networks around a target region. A handful of independent parameters such as the magnitude, source-to-site distance, source mechanisms, travel path, and local site effects^31,32^, are used in these calculations. The methods are being revised and improved, generally incorporating additional parameters to match the equation with the observed values by regression analysis. As a result, a number of equations have been proposed for various regions with different tectonic settings. However, to systematically estimate the PGV for different regions around the world, the present study uses a relatively simple empirical equation presented by Si and Midorikawa^33^, which is derived from data collected in Japan. Because most of the volcanoes and large earthquakes are located around subduction zones, the empirical equation for Japan is considered to be representative of strong ground motion in the cases considered in this study.

The PGV used in the present study is given by

$${\text{log}}_{10} V = 0.58M_{w} + 0.038D + d - 1.29 - {\text{log}}_{10} \left( {X + 0.0028 \times 10^{{0.5M_{w} }} } \right) - 0.02X,$$

where $V$ represents the PGV (cm/s) in a hard medium with an S-wave velocity of 600 m/s, $D$ (km) is the source depth of the earthquake, and $X$ (km) is the minimum distance from the earthquake fault. Additionally, the parameter $d$ is a coefficient that describes the earthquake fault type: $d=0$ for earthquakes in the crust, $d=-0.02$ for inter-plate earthquakes, and $d=0.12$ for intra-plate earthquakes. Because the difference in PGV is only 1.4 cm/s for different values of $d$, $d=0$ is set in the analysis. The fault length ${L}_{f}$ is calculated as ${L}_{f}=\sqrt{A/\pi }$, where *A* is the slip area (km^2^) determined from ${\mathrm{log}}_{10}A=1.02{M}_{w}-4.0$^34^. When a volcano is located within a distance of ${L}_{f}$, *X* was set to 0 km.

## Supplementary Information

Supplementary Information.
