# Supplementary material for: Volcanic eruptions are triggered in static dilatational strain fields generated by large earthquakes
Source: Sci Rep. 2021 Aug 26;11:17235. doi: 10.1038/s41598-021-96756-z (PMC8390651; doi:10.1038/s41598-021-96756-z)
Supplement: Supplementary file 1 — Supplementary Information. [file 41598_2021_96756_MOESM1_ESM.pdf]

Supplementary Figures

Volcanic eruptions are triggered in static dilatational strain fields generated by large earthquakes

Takeshi Nishimura

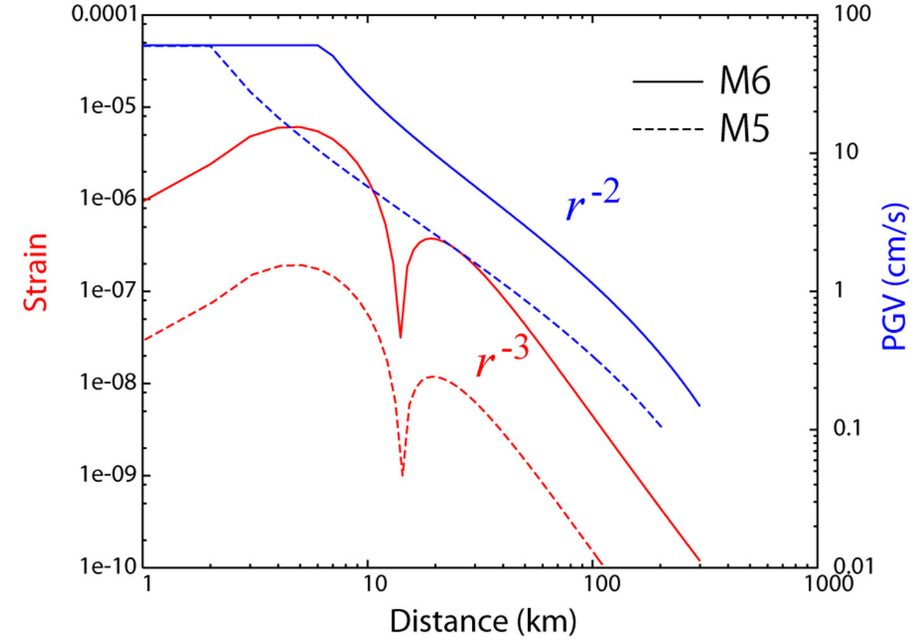

Figure 1. Distance dependence of volumetric strain and PGV for earthquakes with magnitudes of 5 and 6. The source mechanism was assumed to be a reverse fault with a dip of 45°, and the plot shows the maximum dilatational volumetric strain.

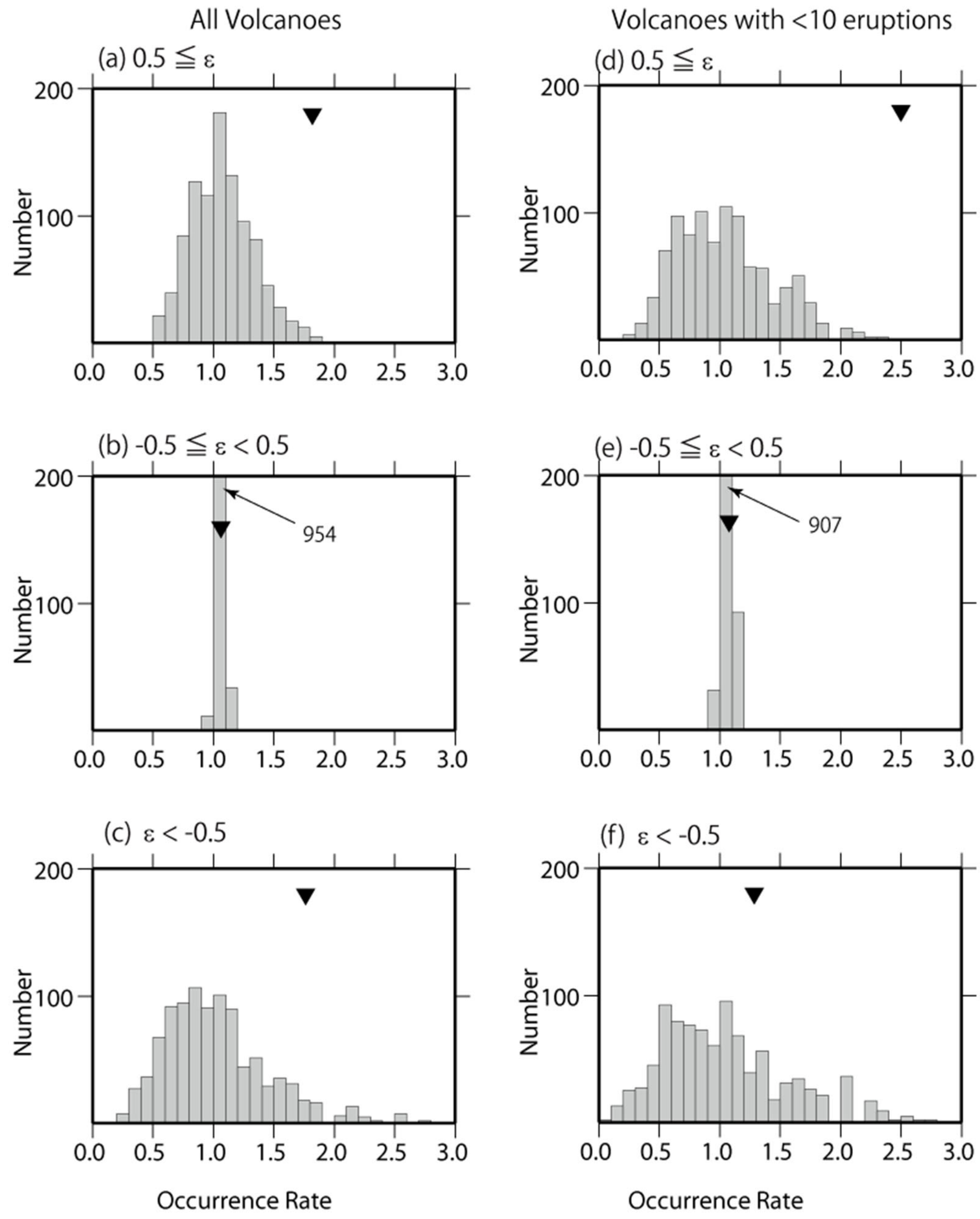

Figure 2. Histograms of eruption occurrence rate for 5 years after large earthquakes obtained from 1000 simulations. The occurrence rates are normalized by the rate 10 years before large earthquakes. Black triangles represent the occurrence rates obtained from the empirical data catalogs. (a)–(c) Results for all volcanoes at different static strains. (d)–(e) Volcanoes that erupted fewer than 10 times between 1966 and 2020. This figure was created by Generic Mapping Tools (GMT) v4.5.5[35].

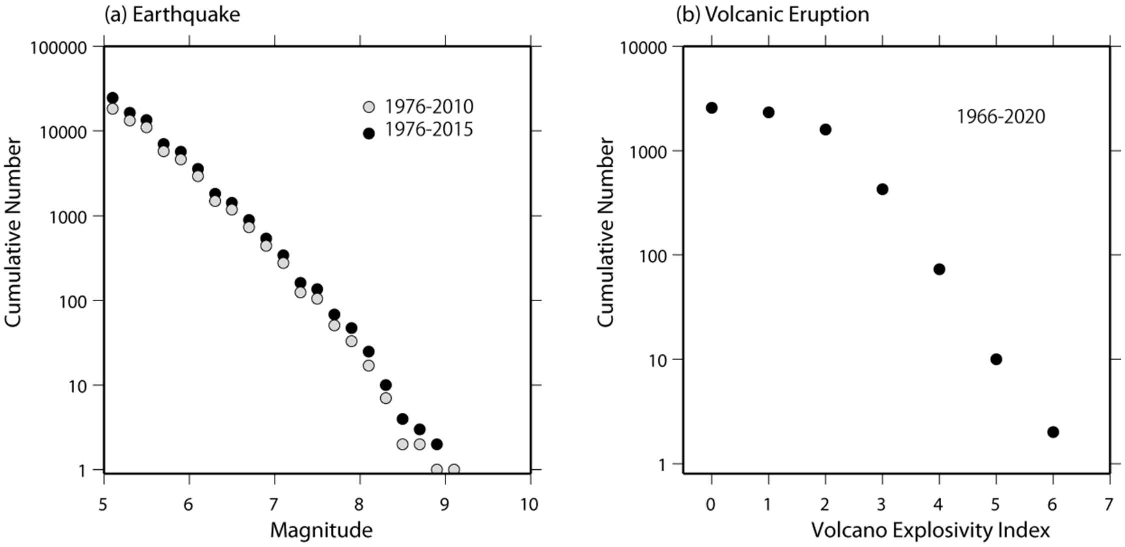

Figure 3. Frequency distributions of (a) earthquake magnitude and (b) VEI. This figure was created by Generic Mapping Tools (GMT) v4.5.5[35].

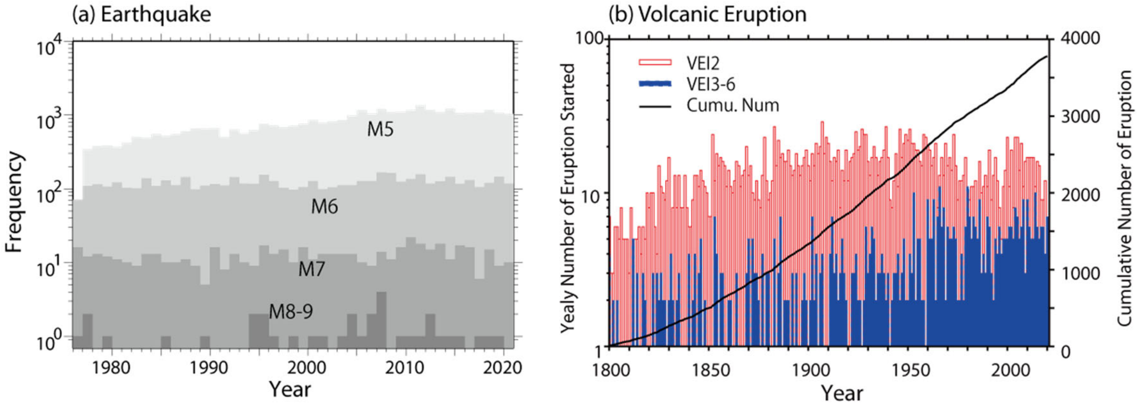

Figure 4. Yearly numbers of earthquakes of M5 and higher and volcanic eruptions with VEIs of 2 or more. The yearly numbers of earthquakes are shown separately for the M5, M6, and M7 classes with numbers in the M8 and M9 classes combined.

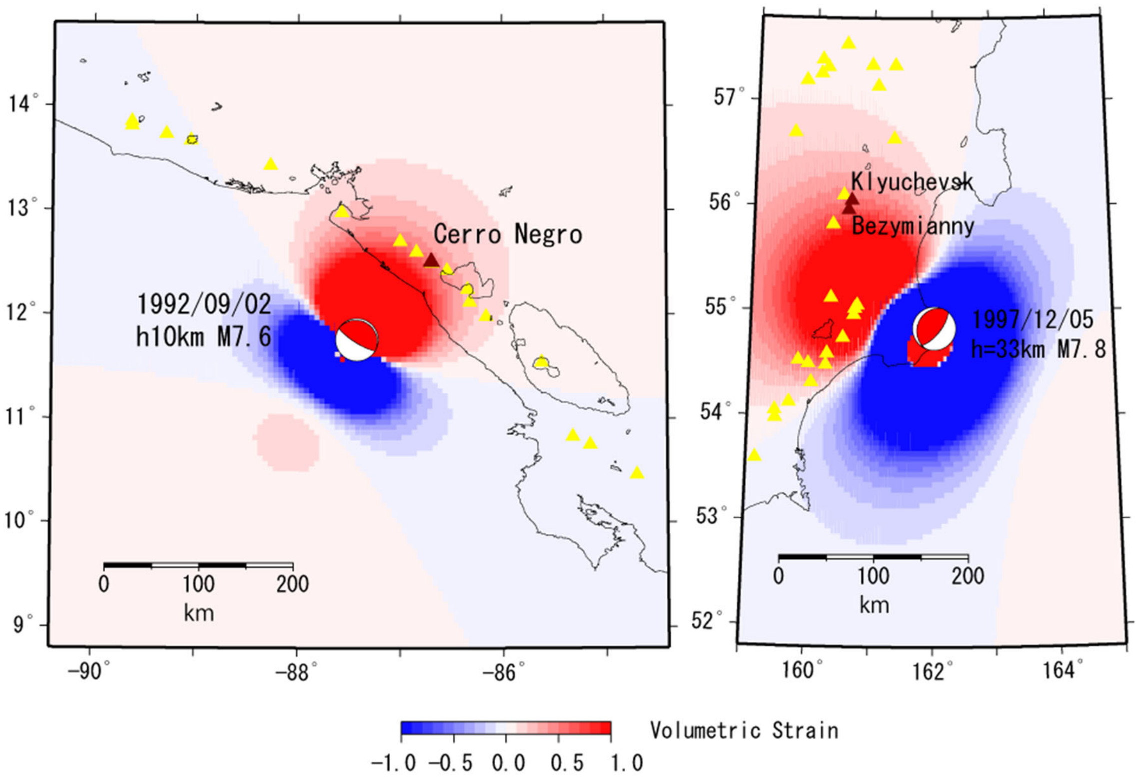

Figure 5. Examples of spatial distribution of volumetric strain caused by large earthquakes. (left) M7.6 earthquake on September 2, 1992. (right) M7.8 earthquake on December 5, 1997. The volcanoes that erupted after the large earthquakes are indicated by brown triangles. Yellow triangles are Holocene volcanoes. This figure was created by Generic Mapping Tools (GMT) v4.5.5[35].

Supplementary Table

Volcanic eruptions are triggered in static dilatational strain fields generated by large earthquakes

Takeshi Nishimura

Table 1. Number of eruptions over 55 years from 1966 to 2020.

Volcano	Number of Eruptions
Bezymianny	35
Klyuchevskoy	27
Etna	26
Ulawun	20
Soputan	19
Raung	18
Ambrym	18
Nyamuragira	17
Lopevi	16
Cleveland	16
Whakaari	15
Pavlof	15
Krakatau	15
Akutan	15
Lascar	14
Karangetang	14
Kanlaon	13
San Cristobal	12
Lokon-Empung	12
Langila	12
Kerinci	12
Bulusan	12
Shishaldin	11
Mayon	11
Gamalama	11
Chikurachki	11
Sheveluch	10
Marapi	10
Llaima	10
Gaua	10
Galeras	10
Fernandina	10
Concepcion	10
Asosan	10

Table 2. Percentages of simulation cases with more eruptions than the observed number. The percentages are shown for different strain ranges (units: micro strain) and the period from the occurrence of large earthquake. Alleruption data for all volcanoes are used. ($N_{er} < 10$) The volcanoes that erupted less than 10 times for 55 years from 1966 to 2020 are listed.

Period (year)	0–5	0–6	0–7	0–8	0–9	0–10
(All data)						
$0.5 \leq \varepsilon$	0.9	0.4	1.0	1.1	0.9	0.7
$-0.5 \leq \varepsilon < 0.5$	34.2	30.3	39.8	51.3	52.4	54.2
$\varepsilon < -0.5$	7.3	10.6	8.5	12.9	15.1	19.0
($N_{er} < 10$)						
$0.5 \leq \varepsilon$	0.6	0.4	0.4	0.6	0.4	0.7
$-0.5 \leq \varepsilon < 0.5$	29.5	19.2	22.0	27.7	31.8	26.5
$\varepsilon < -0.5$	29.9	35.9	39.7	50.5	53.8	63.1

Table 3. List of large earthquakes and volcanic eruptions. Y, M, and D represent the year, month, and day of occurrence. Lon and Lat are the longitude and latitude of earthquakes in degrees. Z [km] is the depth. M is the earthquake magnitude. Nv represents the number of volcanoes for which the static dilatational strain exceeded 0.5 μ, and Nb and Na are the numbers of eruptions occurring 10 years before or after the earthquake, respectively, at those volcanoes. T represents the occurrence time of the eruption minus that of the large earthquake. D [km] is the distance from the earthquake to the volcano, and the strain and PGV are the static strain (units: micro strain) and peak ground velocity [cm/s]. Earthquakes without any volcanic eruptions in the right-hand column are those that introduced a static dilatational strain exceeding 0.5 μ with no eruption.

Earthquake										Volcanic Eruption								
Y	M	D	Lon.	Lat.	Z	M	Nv	Nb	Na	Y	M	D	VEI	T	D	Strain	PGV	Volcano
1976	1	14	−177.9	−29.2	69	7.8	1	0	0									
1976	8	16	124.0	6.3	33	8.0	1	0	0									
1978	3	23	148.4	44.9	33	7.6	2	0	0									
1979	2	28	−141.6	60.6	15	7.4	1	0	0									
1979	12	12	−79.4	1.6	24	8.1	3	0	1	1989	2	19	2	9.2	225	0.66	8.8	Galeras
1980	1	1	−27.8	38.8	10	6.9	1	0	0									
1982	1	12	−87.6	13.2	4	6.1	1	0	0									
1983	5	26	139.2	40.4	33	7.7	4	0	0									
1983	11	16	−155.5	19.4	11	6.6	1	0	0									
1985	3	3	−72.0	−33.2	33	7.9	1	1	1	1987	11	28	2	2.7	205	0.50	7.9	Tupungatito
1985	9	19	−102.6	18.2	33	8.0	1	0	0									
1986	5	7	−174.8	51.4	33	7.9	4	0	1	1987	3	4	2	0.8	117	1.12	24.7	Korovin
1986	8	14	126.6	1.8	43	7.5	1	0	0									
1986	11	14	121.8	24.0	33	7.3	1	0	0									
1987	3	6	−77.8	0.2	12	7.1	1	0	0									
1989	6	26	−155.1	19.4	9	6.4	1	1	0									
1990	3	25	−84.9	9.9	19	7.3	2	1	1	1994	3	16	2	4	80	0.52	13.1	Poas
1991	12	22	151.1	45.5	26	7.6	1	0	0									

1992	9	2	−87.4	11.8	10	7.6	5	1	5	1999	5	1	2	6.7	114	0.75	11.6	San
										1994	7	31	2	1.9	113	0.78	11.7	Telica
										1999	5	21	2	6.7	113	0.78	11.7	Telica
										1995	5	29	2	2.7	114	0.73	11.5	Negro Cerro
										1999	8	5	2	6.9	114	0.73	11.5	Negro Cerro
1993	7	12	139.3	42.8	17	7.7	1	0	1	2000	3	31	2	6.7	135	0.89	10.9	Toya
1994	2	15	104.2	−5.1	15	6.8	1	0	0									
1994	6	6	−76.1	2.9	9	6.8	1	0	0									
1994	10	4	147.3	43.7	33	8.3	4	0	0									
1995	4	7	−173.6	−15.2	31	7.3	1	0	0									
1995	10	9	−104.2	19.2	49	8.0	1	1	1	1997	11	22	3	2.1	75	1.28	95.9	Colima
1995	12	3	149.3	44.6	22.1	7.9	3	1	0									
1996	6	10	−177.6	51.6	33	7.9	1	0	0									
1997	12	5	162.0	54.8	33	7.8	7	12	20	1997	12	5	3	0	156	0.68	11.5	Bezymianny
										1999	2	25	2	1.2	156	0.68	11.5	Bezymianny
										2000	3	14	2	2.3	156	0.68	11.5	Bezymianny
										2000	7	18	2	2.6	156	0.68	11.5	Bezymianny
										2001	7	23	3	3.6	156	0.68	11.5	Bezymianny
										2001	12	10	2	4	156	0.68	11.5	Bezymianny
										2002	12	25	2	5.1	156	0.68	11.5	Bezymianny
										2003	7	26	3	5.6	156	0.68	11.5	Bezymianny
										2004	1	14	3	6.1	156	0.68	11.5	Bezymianny
										2005	11	29	2	8	156	0.68	11.5	Bezymianny
										2006	4	16	3	8.4	156	0.68	11.5	Bezymianny
										2007	5	10	3	9.4	156	0.68	11.5	Bezymianny
										1998	7	23	2	0.6	162	0.58	10.6	Klyuchevskoy

										1999	2	5	2	1.2	162	0.58	10.6	Klyuchevskoy
										2000	2	3	2	2.2	162	0.58	10.6	Klyuchevskoy
										2000	7	28	2	2.6	162	0.58	10.6	Klyuchevskoy
										2002	4	9	2	4.3	162	0.58	10.6	Klyuchevskoy
										2002	11	24	2	5	162	0.58	10.6	Klyuchevskoy
										2005	1	20	2	7.1	162	0.58	10.6	Klyuchevskoy
										2007	2	15	2	9.2	162	0.58	10.6	Klyuchevskoy
1998	11	9	129.0	−6.9	33	7.0	1	0	0									
1999	2	13	144.8	−3.6	10	6.2	1	0	0									
1999	11	26	168.2	−16.4	33	7.4	1	1	6	2001	6	8	3	1.5	18	7.02	95.6	Lopevi
										2003	6	8	3	3.5	18	7.02	95.6	Lopevi
										2004	9	16	2	4.8	18	7.02	95.6	Lopevi
										2005	1	30	2	5.2	18	7.02	95.6	Lopevi
										2005	10	27	2	5.9	18	7.02	95.6	Lopevi
										2007	4	21	2	7.4	18	7.02	95.6	Lopevi
										2008	2	24	2	8.3	18	7.02	95.6	Lopevi
2001	1	13	−88.7	13.1	60	7.7	4	0	0									
2001	6	23	−73.6	−16.3	33	8.4	3	1	2	2003	7	30	2	2.1	198	2.80	25.4	Sabancaya
										2006	3	25	2	4.8	293	0.58	9.5	Ubinas
2002	3	5	124.3	6.0	31	7.5	1	0	0									
2003	1	22	−104.1	18.8	24	7.5	1	1	1	2013	1	6	2	10	96	1.01	14.6	Colima
2003	9	25	143.9	41.8	27	8.3	4	1	0									
2003	11	17	178.7	51.2	33	7.7	1	0	0									
2004	12	26	95.8	3.3	10	9.0	4	1	2	2010	8	27	2	5.7	290	1.52	42.0	Sinabung
										2013	9	15	4	8.7	290	1.52	42.0	Sinabung
2005	3	28	97.1	2.1	30	8.6	2	0	2	2010	8	27	2	5.4	186	5.70	49.7	Sinabung

										2013	9	15	4	8.5	186	5.70	49.7	Sinabung
2006	5	3	−174.1	−20.2	55	8.0	6	0	3	2009	3	17	2	2.9	137	0.89	27.1	Hunga
										2014	12	19	2	8.6	137	0.89	27.1	Hunga
										2006	8	7	2	0.3	150	0.58	22.6	Home
2006	11	15	153.3	46.6	38.9	8.3	8	0	1	2009	6	11	4	2.6	169	1.14	30.0	Sarychev
										2010	9	20	2	3.8	169	1.14	30.0	Sarychev
2007	1	21	126.3	1.1	22	7.5	1	1	3	2011	12	5	2	4.9	121	0.60	9.6	Gamalama
										2014	12	18	2	7.9	121	0.60	9.6	Gamalama
										2015	7	16	2	8.5	121	0.60	9.6	Gamalama
2007	9	12	101.4	−4.4	34	8.5	3	0	0									
2007	9	12	100.8	−2.6	35	7.9	2	5	2	2013	6	2	2	5.7	112	2.29	27.4	Kerinci
										2016	3	31	2	8.6	112	2.29	27.4	Kerinci
2008	4	16	−179.2	51.9	13	6.6	1	0	0									
2008	6	13	140.9	39.0	7.8	6.9	1	0	0									
2010	2	27	−72.7	−35.9	44.8	8.8	10	1	6	2010	9	6	2	0.5	206	9.41	80.5	Planchon–Peteroa
										2011	2	17	3	1	206	9.41	80.5	Planchon–Peteroa
										2016	1	8	2	5.9	164	9.45	137.1	Nevados de Chillan
										2012	12	22	2	2.8	261	1.01	40.7	Copahue
										2015	9	18	2	5.6	261	1.01	40.7	Copahue
										2019	8	2	2	9.4	261	1.01	40.7	Copahue
